# Prevalence of rs850683722 Variant and Its Influence on the Course of Myxomatous Mitral Valve Disease in 105 Cavalier King Charles Spaniel Dogs in the Polish Population

**DOI:** 10.3390/ani16131956

**Published:** 2026-06-24

**Authors:** Maksymilian Lewicki, Sylwia Barbara Górczyńska-Kosiorz, Justyn Gach, Piotr Frydrychowski, Zuzanna Wojtczak, Agnieszka Noszczyk-Nowak

**Affiliations:** 1 Department of Internal Medicine and Clinic of Diseases of Horses, Dogs and Cats, Wrocław University of Environmental and Life Sciences, Grunwaldzki Sq. 47, 50-366 Wrocław, Poland; justyn.gach@upwr.edu.pl (J.G.); piotr.frydrychowski@upwr.edu.pl (P.F.); zuzanna.sidoruk@upwr.edu.pl (Z.W.); 2 Department of Internal Medicine, Diabetology and Nephrology, Faculty of Medical Sciences in Zabrze, Medical University of Silesia, 40-055 Katowice, Poland; skosiorz@sum.edu.pl

**Keywords:** CKCS, MMVD, ACE, polymorphism, NGS, Sanger, RAAS

## Abstract

Myxomatous mitral valve disease (MMVD) is the most common acquired cardiac disease in small-breed dogs and shows particularly high prevalence and early onset in Cavalier King Charles Spaniels (CKCS). Although MMVD is considered a complex, polygenic disease, the clinical relevance of individual genetic variants remains incompletely understood. The angiotensin-converting enzyme (ACE) gene variant rs850683722 has previously been associated with altered ACE activity and differences in renin–angiotensin–aldosterone system-related responses in dogs with MMVD. The aim of this study was to determine the prevalence of rs850683722 in a Polish population of CKCS dogs and to assess whether this variant is associated with the clinical course of MMVD. A total of 105 CKCS dogs were included in the study. All dogs underwent standardized cardiovascular evaluation, including echocardiography, electrocardiography, and systolic blood pressure measurement. MMVD diagnosis and staging were performed according to current ACVIM consensus criteria. Genotyping of the rs850683722 variant was performed using Sanger sequencing for 95 dogs, while next-generation sequencing data was obtained for 10 dogs. Genotype distribution, allele frequencies, conformity with the Hardy–Weinberg equilibrium (HWE), sex-related differences, and associations between genotype and age at progression to selected MMVD stages or the primary clinical endpoint were assessed statistically. The most frequent genotype was AA, detected in fifty-nine dogs, followed by GG in thirty-seven dogs and AG in nine dogs. When dogs carrying at least one A allele were considered variant-positive, the overall prevalence of the variant-positive genotype was 64.8%. The calculated allele frequencies were 0.605 for the A allele and 0.395 for the G allele. The observed genotype distribution deviated markedly from the Hardy–Weinberg equilibrium, mainly because of a pronounced deficit of heterozygous dogs. No significant association was detected between genotype and sex. Genotype was also not significantly associated with age at progression to stage B2 or stage C. A statistically significant difference in age of death was demonstrated by genotype, but this difference was not reflected in the survival analysis. The rs850683722 variant was highly prevalent in the studied Polish CKCS population, with a frequency comparable to previously reported data for this breed. Despite its documented biological association with ACE activity and RAAS-related responses, the variant was not significantly associated with the clinical progression of MMVD in this cohort. These findings suggest that rs850683722 alone seems unlikely to be a reliable marker for predicting the severity or rate of MMVD progression in Polish CKCS dogs. Further studies including larger cohorts, longer follow-up, pedigree information, and the direct assessment of RAAS activity may help clarify whether this variant has stage-dependent or treatment-related clinical relevance.

## Simple Summary

Myxomatous mitral valve disease is the most common acquired heart disease in small-breed dogs and is particularly frequent in Cavalier King Charles Spaniels. Genetic factors are believed to contribute to the early development and progression of this disease, but the clinical significance of individual variants remains uncertain. This study evaluated the frequency of a variant in the angiotensin-converting enzyme gene, rs850683722, in 105 Cavalier King Charles Spaniels from Poland and assessed whether this variant was associated with the clinical course of myxomatous mitral valve disease. The variant was common in the studied population, and its frequency was similar to that reported previously in Cavalier King Charles Spaniels from other populations. However, no significant association was found between genotype and the age at progression to stages B2 or C. A statistically significant difference in age of death by genotype was demonstrated, but this was not reflected in the survival analysis. These findings suggest that although this variant may influence renin–angiotensin–aldosterone system activity, it does not appear to be a reliable standalone marker for predicting the clinical progression of myxomatous mitral valve disease in Cavalier King Charles Spaniels.

## 1. Introduction

Myxomatous mitral valve disease (MMVD) is the most common heart disease in small and miniature breeds of dogs. The Cavalier King Charles Spaniel (CKCS) is particularly susceptible to this condition, with a prevalence of up to 100% after the age of eight depending on studied cohorts. MMVD is known to be a polygenetic disease with several genetic variants being suspected to induce earlier development of the disease [1,2,3,4,5,6,7,8,9,10,11,12,13,14].

The ACVIM classification with the support of MINE Score2 divides MMVD into stages, ranging from breed predisposition to advanced, refractory heart failure, and provides guidelines for diagnosis and treatment at specific stages of the disease [1,2,6,12,15,16,17,18,19]. Stage A includes dogs of predisposed breeds without detectable clinical or echocardiographic changes, while stage B consists of animals diagnosed with mitral valve disease but without signs of congestive heart failure. Stage B includes stage B1, in which significant cardiomegaly is not observed, and only clinical monitoring and periodic echocardiographic evaluation are usually recommended, and stage B2, characterized by cardiac remodeling, in which the administration of pimobendan is recommended to delay the onset of heart failure. Stage C includes dogs with current or previous congestive heart failure secondary to MMVD and requires combined treatment, primarily with a loop diuretic, pimobendan, and usually an angiotensin-converting enzyme inhibitor and/or a mineralocorticoid receptor antagonist. Stage D is defined as heart failure refractory to standard therapy, requiring individualized intensification of treatment, including increased doses of diuretics, sequential diuretic therapy, optimization of renin–angiotensin–aldosterone system blockade, and treatment of co-occurring complications such as pulmonary hypertension or cardiac arrhythmias.

Canine MMVD is currently understood as an active, cell-mediated degenerative valvulopathy rather than a passive senescent lesion, in which quiescent valvular interstitial cells undergo phenotypic conversion into activated myofibroblast- and mesenchymal-like cells, with increasing cellularity, matrix-remodeling activity, proteoglycan/glycosaminoglycan accumulation in the spongiosa, attenuation and disorganization of the collagen-rich fibrosa, and progressive loss of normal leaflet biomechanics, ultimately leading to leaflet thickening, prolapse, and mitral regurgitation [20,21,22,23,24,25].

Developmental reprogramming and endothelial-to-mesenchymal transition (EndoMT) appear to contribute significantly to this process, as diseased valves show basement-membrane disarray, together with the altered expression of ACTA2, SNAI1, CDH5, NOTCH1, and other developmental and mesenchymal markers, consistent with the reactivation of embryonic valvulogenic pathways in adult valve tissue [22,25].

At the molecular level, a serotonin–TGF-β signaling axis remains one of the best-supported mechanisms of primary valvular degeneration because myxomatous valves exhibit increased local serotonin synthetic capacity and 5-HT2B receptor expression, reduced serotonin transporter expression, increased ERK1/2 activation, and enhanced TGF-β signaling, while more recent in vitro canine studies indicate that TGF-β-driven PI3K/AKT/mTOR activation sustains the diseased valvular interstitial-cell phenotype through the effects on myofibroblast differentiation, senescence, apoptosis, autophagy, and the secretory profile of activated cells [21,26,27,28].

Degenerative change also extends beyond the leaflets to the subvalvular apparatus: recent work by Gach et al. demonstrated that chordae tendineae affected by MMVD show marked extracellular-matrix disorganization involving collagens I, III, and IV; fibronectin; chondroitin; and tenascin, together with progressive biomechanical weakening and greater susceptibility to rupture, indicating that chordal pathology is an integral component of disease progression [29].

By contrast, the role of the renin–angiotensin–aldosterone system (RAAS) in primary leaflet initiation appears less direct because an autoradiographic study did not demonstrate meaningful ACE or angiotensin II receptor expression in canine myxomatous mitral leaflets, arguing against RAAS as a dominant trigger of the initial valvular lesion [30].

Nevertheless, as regurgitation becomes hemodynamically relevant, RAAS assumes major importance as a progression-amplifying neurohormonal pathway. Neurohormonal activation promotes maladaptive cardiac remodeling and congestion. Dogs with preclinical stage B2 MMVD show an altered circulating RAS profile characterized particularly by increased alternative RAS/ACE2 activity rather than uniform elevation of the classical pathway. Aldosterone breakthrough is common despite ACE-inhibitor therapy, and sequential RAAS blockade can improve remodeling even when it does not clearly delay the onset of overt heart failure [1,2,16,17,31,32,33,34,35].

In advanced MMVD, secondary myocardial remodeling extends beyond ventricular adaptation and includes distinct changes within the left atrial myocardium. Janus et al. [36] demonstrated that dogs with end-stage MMVD and severe left atrial enlargement showed an altered immunohistochemical expression of markers related to cardiomyocyte structure, mesenchymal/interstitial activation, extracellular matrix remodeling, and apoptosis. Irregular desmin cross-striation and reduced desmosomal staining indicated the cytoskeletal disorganization of atrial cardiomyocytes, while vimentin-positive interstitial cells and altered periostin localization supported the involvement of fibroblast-associated extracellular matrix remodeling. The observed changes in caspase-3 expression further suggest that apoptosis-related pathways may participate in the remodeling process, although they appear less prominent in MMVD than in dilated cardiomyopathy. These findings indicate that in late-stage MMVD, left atrial enlargement is accompanied by true myocardial and interstitial remodeling rather than representing a purely passive consequence of chronic mitral regurgitation [36].

Taken together, the available literature supports a model in which MMVD is initiated primarily by valvular interstitial-cell activation, EndoMT, and serotonin/TGF-β-dependent extracellular-matrix remodeling, whereas RAAS acts predominantly as a systemic maladaptive amplifier of remodeling and clinical progression once chronic mitral regurgitation is established.

The genetic basis of MMVD is not yet well understood. The available literature describes individual loci or regions that are potentially responsible for a more severe course of MMVD, such as variants within the *NEBL* gene, but data are still very sparse [8,37,38,39,40,41,42,43].

One variant of potential clinical significance within the angiotensin-converting enzyme gene is the rs850683722 variant [44,45]. Relative to the CanFam3.1 reference sequence, this is an intronic variant at position 9:11507816:G>A. According to the current reference sequence, the coordinate location of this variant has changed, and the reference and alternative alleles have also been inverted; relative to the ROS_Cfam_1.0 sequence, this is 9:13171340:A>G. Current knowledge suggests a possible regulatory effect of this variant, but the exact molecular mechanism has not been clearly described. However, the biological effect for this variant has been identified. In the study by Meurs et al. 2017, in dogs with MMVD, dogs positive for the variant had significantly lower ACE activity before treatment with an ACE inhibitor, in contrast to wild-type dogs [33]. After 2 weeks of enalapril treatment, ACE activity decreased in both groups, and the final ACE activity did not differ significantly between genotypes. In a subsequent study by Meurs et al. in 2018, focusing exclusively on CKCS with MMVD, 73 dogs were evaluated. Median ACE activity was lower in dogs with the variant than in wild-type dogs [34]. Adin et al., 2020, which assessed the RAAS metabolite profile before and after enalapril in dogs with MMVD, found no significant differences in RAAS activity between variant-positive and variant-negative dogs [35]. After enalapril, the classic RAAS axis was inhibited and the alternative axis was enhanced in both genotypes, but aldosterone breakthrough occurred more frequently in variant-positive dogs, and aldosterone concentrations after enalapril were significantly higher in variant-positive dogs.

Published data clearly demonstrate the high prevalence of the rs850683722 variant in certain dog breeds, particularly in CKCS, as well as its impact on angiotensin-converting enzyme activity, the RAAS profile following ACE inhibitor administration, and the occurrence of aldosterone crisis. Most data on the prevalence of this variant are from North American dogs, with one study partially based on a Danish dog population [34]. However, there is a lack of population-based data on the prevalence of this variant for other European countries, including Poland. No data describing differences in the course of MMVD depending on the occurrence of the rs850683722 variant have been published so far, which leaves room for further research verifying the real impact of this variant on the course of MMVD and geographical differences in its occurrence.

Considering the role of RAAS in the pathogenesis of MMVD, especially in its later stages, the documented effect of the rs850683722 variant on RAAS activity may potentially modulate the course of MMVD.

The aim of this study was to assess the prevalence of the rs850683722 variant in the Polish population of CKCS dogs and its influence on the course of MMVD.

## 2. Materials and Methods

### 2.1. Study Group and Inclusion Criteria

The study was conducted at the Department of Internal Medicine and Clinic of Diseases of Horses, Dogs, and Cats, Wrocław University of Environmental and Life Sciences, Grunwaldzki Sq. 47, 50-366 Wrocław, Poland, and “GLIWICKA PRZYCHODNIA WETERYNARYJNA” Toszecka 19 str. Gliwice, Poland. CKCS dogs were prospectively enrolled for this study between 27 March 2023 and 10 March 2025. Clinical monitoring was conducted until May 2026. The primary endpoint was death of the animal due to congestive heart failure or entry into stage D with non-standard treatment (loop diuretic doses above the permissible limits, use of sacubitril/valsartan, etc.). The study inclusion criteria included the following: breed—CKCS, age—over 1 year (younger dogs were enrolled only if they showed signs of MMVD during examination; MMVD needed to be excluded in earlier examination performed by a nationally recognized specialist), diagnosis of MMVD, no congenital heart defects, and no diseases or treatment directly affecting the RAA system. Dogs with congenital heart defects, other cardiovascular disorders such as dilated cardiomyopathy phenotype or heart tumors, systemic conditions with the possibility of influencing RAAS activity (e.g., renal disease, endocrine disorders, neoplasia), or receiving medications that are known to affect the renin–angiotensin–aldosterone system (e.g., ACE inhibitors, mineralocorticoid receptor antagonists, glucocorticosteroids) were excluded from the study. At the recruitment stage, dogs in stages A to C were accepted for the study; for dogs in stage C, the condition was not to have previously taken drugs from the group of ACE inhibitors and mineralocorticoid receptor antagonists.

The minimum acceptable sample size was estimated for the primary objective of determining the frequency of the rs850683722 variant in the CKCS study population. Using a conservative expected variant frequency of 50%, a 95% confidence level, and an acceptable precision of ±10 percentage points, the minimum required sample size was 96 dogs.

### 2.2. Cardiovascular Examination, MMVD Diagnosis, and Treatment

Each dog underwent a standardized examination protocol that included echocardiography (Philips Ultrasound Inc. EPIQ Elite, Bothell, WA, USA; GE Vingmed Ultrasound AS, VIVID S70n, Horten, Norway), 12-lead resting electrocardiography (BTL Industries Ltd., BTL-08 MT Plus, Stevenage, UK) with electrode placement according to Santilli et al., and arterial systolic blood pressure measurement using the Doppler method (Parks Medical Electronics Inc., model 811-B, Aloha, OR, USA) [46]. The echocardiographic measurement protocol included the following: M mode study of the left and right ventricles with normalization of left ventricular diameters, Simpson disk method measurement of the left ventricle in right parasternal four-chamber long-axis view and left four-chamber apical view (at least one), and PW and CW Doppler assessment of the mitral, tricuspid, aortic, and pulmonic flows. The atrial measurements included LA/Ao ratio and LAD measurement [47,48,49,50]. The diagnosis of MMVD stage was placed in accordance with the latest ACVIM consensus statement criteria. MMVD treatment for each dog was also performed according to the recommendations described in the ACVIM consensus. For stages A and B1, no treatment was administered, with follow-up visits every 6 months. For stage B2, pimobendan was given at a dose of 0.25–0.3 mg/kg every 12 h, with follow-up visits every 3 months. For stage C, pimobendan was given at a dose of 0.25–0.3 mg/kg every 12 h, along with a loop diuretic—furosemide or torasemide at the lowest effective dose—with dose titration during progression up to 8 mg/kg/day for furosemide or 0.6 mg/kg/day for torasemide; benazepril at a dose of 0.25 mg/kg every 24 h; and spironolactone at a dose of 2 mg/kg every 24 h, with follow-up visits every 3 months or more frequently if symptoms worsened or there was suspicion of earlier decompensation [1,2,6,15,16,17].

### 2.3. DNA Sampling

**Sanger sequencing**

The DNA sequencing using Sanger’s method was performed using the same starters as Meurs K. et al. in their original studies [44]. The forward primer was 5′-TCAGCTCCATGCAATCCATA-3′; the reverse primer was 5′-CCCCTTGCCCTATCTGTAAA-3′.

For each sequencing reaction, 3 μL of BigDye™ Terminator v3.1 Ready Reaction Mix, 1 μL of BigDye™ Terminator v1.1 and v3.1 5X Sequencing Buffer (Thermo Fisher Scientific Baltics, Vilnius, Lithuania), 5 pmol of the appropriate primer, and 50–250 ng of DNA template were mixed in a final 10 μL volume.

Cycle sequencing was performed as follows in 100 μL PCR tubes. Incubation at 96 °C for 1 min was performed as the initial denaturation step, followed by 25 cycles of 96 °C for 10 s, 54 °C for 5 s, and 60 °C for 4 min of incubation. Prior to purification, the reaction mix was incubated at 4 °C. Purified reaction products were separated by electrophoresis on the 3730xl DNA Analyzer according to the manufacturer’s references (Thermofisher, Waltham, MA, USA).

After obtaining the results from the external laboratory, all obtained sequences were additionally manually verified using Finch TV 1.4.0 software against the variant and the surrounding sequence.

For 10 dogs, data on variant occurrence were obtained using NGS sequencing.

**Next-generation sequencing methodology**

Library preparation and sequencing were performed on an Illumina NovaSeq series instrument in 2 × 150 nt mode with 25–30× coverage. Raw reads (FASTQ) were subjected to initial quality control and bioinformatics processing according to standard NGS data analysis practices.

Reads were trimmed to remove adapters and low-quality ends using Cutadapt v2.8.

Trimmed reads were mapped to the dog reference genome (ROS_Cfam_1.0, GCF_014441545.1) using the Burrows–Wheeler Aligner algorithm (BWA-MEM v0.7.15-r1140).

The resulting BAM files were sorted using Picard v2.20.4. Duplicate reads in the BAM file were located and marked using the MarkDuplicates module from the Picard package.

Base quality score recalibration (BQSR) was performed using the Genome Analysis Toolkit (GATK) v4.2.2.0.

Detection of SNP and indel variants within the ACE gene (as well as detection of variants located up to 1500 nucleotides upstream of the ACE gene) was performed using the HaplotypeCaller tool from the GATK v4.2.2.0 package. The analysis was conducted according to the recommended GATK Best Practices workflow.

The resulting VCF files were normalized using bcftools v1.6, which included the decomposition of multiallelic variants and left alignment of indels relative to the reference genome.

The final set of variants was functionally annotated using the Variant Effect Predictor (VEP) tool—ROS_Cfam_1.0, which allowed for the determination of the potential impact of the variants on the genes.

The analyzed region included the complete *ACE* gene sequence according to ROS_Cfam_1.0, together with up to 1.5 kb upstream of the gene.

### 2.4. Statistical Analysis

Statistical testing included assessing the acquired dog cohort for normal age at the inclusion distribution. The study population was assessed for observed allele distribution conformance to the expected values according to the Hardy–Weinberg law using the Chi square test [51,52]. Genotype versus age at onset of stages B2 and C and age at death or stage D were also analyzed using Kruskal–Wallis and Mann–Whitney tests. The Kruskal–Wallis test was used to compare continuous variables between three independent genotype groups, AA, AG, and GG, if all three genotype categories contained evaluable observations for a given endpoint. The Mann–Whitney U test was used when only two independent groups were compared, including analyses comparing variant-positive dogs, defined as AA/AG, with homozygous wild-type dogs, defined as GG, or endpoint-specific analyses in which observations contained only two genotype categories. Kaplan–Meier survival curves were generated for patients stratified by genotype based on the achievement of endpoints and then compared using the log-rank Mantel–Cox test. Statistical testing was performed using Statistica 13.3, GraphPad 5.03, and Past 5.3 software.

## 3. Results

One hundred and five dogs met the study inclusion criteria.

In the study group, 50 individuals were females and 55 were males.

**Figure 1:**
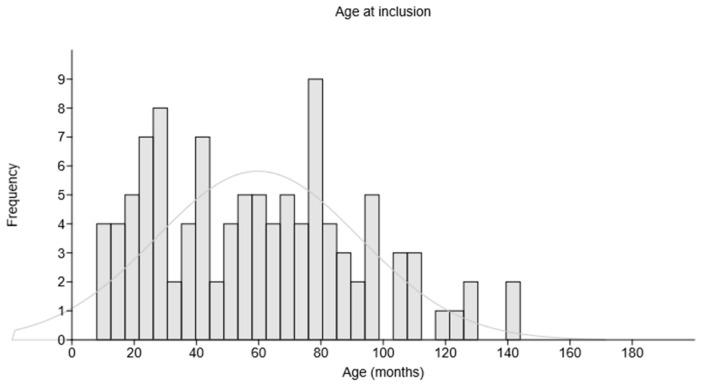
Histogram showing the age distribution of the study population on the day of enrollment in the study with a normal distribution curve superimposed.

The study population, in terms of age (months) at the time of inclusion in the project, deviated from the normal distribution with a dominance of relatively young individuals (Shapiro–Wilk W = 0.9657; *p* (normal) = 0.00803; Min = 8; Max = 144; Mean = 59.75; Stand. dev = 32.6381; Median = 58; Q1–Q3 of 29.5–80.5; shown in Figure 1)).

During the clinical monitoring period, 13 dogs reached the primary endpoint—death. As of 25 May 2026, which was the end of observation for this study, forty-nine dogs were in stage B1, eleven in stage B2, and nine in stage C. Contact was lost with 23 dogs, representing 21.9% of the studied cohort (Table 1). A summary table showing the dates of birth, genotypes of the individuals, and their current clinical status is provided in Supplementary Materials.

**Figure 2:**
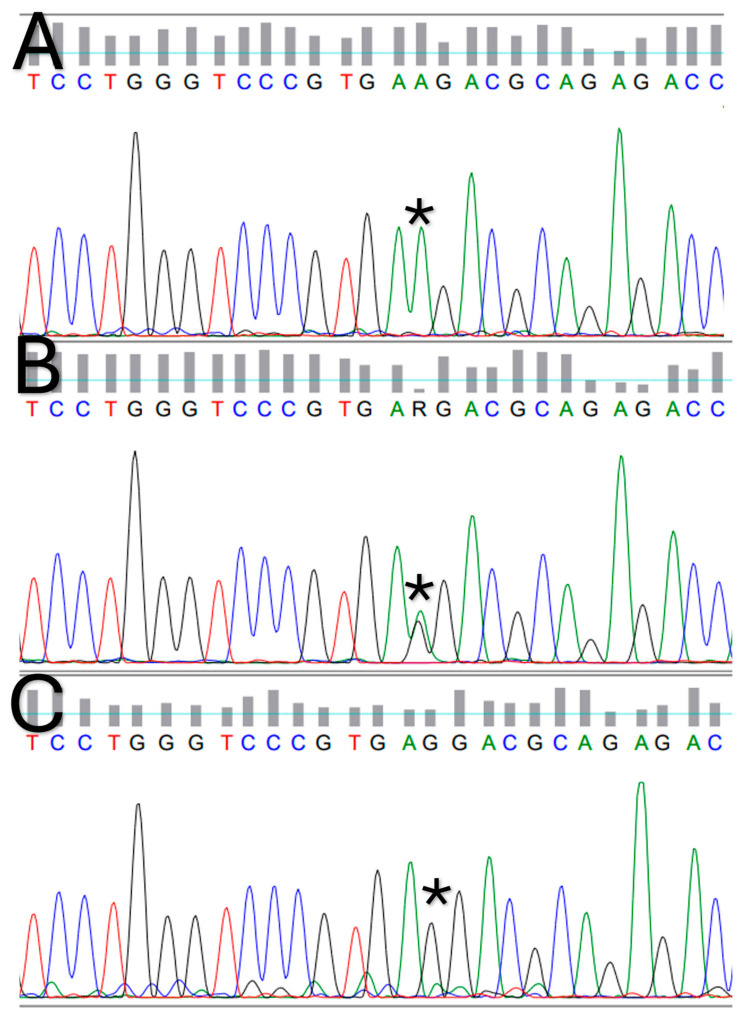
Sequence surrounding the rs850683722 variant in the sample individuals. The forward sequence variant is marked with an asterisk. (**A**)—homozygote AA, (**B**)—heterozygote, (**C**)—homozygote GG.

In the analyzed cohort, the most frequent genotype was AA, identified in 59 dogs, representing 56.2% of the population. The GG genotype was found in thirty-seven dogs, corresponding to 35.2%, whereas the heterozygous AG genotype was detected in nine dogs, corresponding to 8.6% (Figure 2). When dogs carrying at least one copy of the A allele were considered jointly, the overall prevalence of the variant-positive genotype, defined as AA + AG, was 64.8%. The calculated allele frequency was 0.605 for the A allele and 0.395 for the G allele.

No significant association was detected between sex and genotype distribution. Among females, twenty-nine dogs were AA, four were AG, and seventeen were GG. Among males, thirty dogs were AA, five were AG, and twenty were GG. The genotype distribution did not differ significantly between sexes, and the dichotomized comparison of variant-positive dogs, defined as AA/AG, versus wild-type homozygous dogs, defined as GG, also showed no significant sex-related difference.

**Figure 3:**
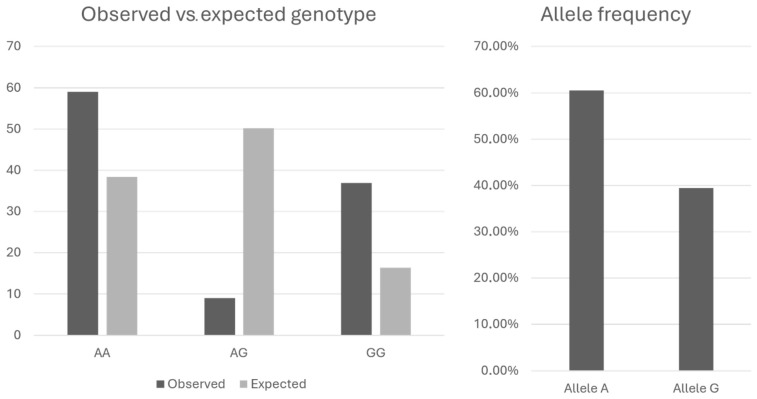
Comparison of observed genotype distribution vs. expected distribution according to Hardy–Weinberg law and allele frequency in studied cohort. Chi2: 41.355; *p* (no assoc.): <0.000001; Fisher’s exact: *p* (no assoc.): <0.000001.

The observed genotype distribution showed a marked deviation from the Hardy–Weinberg equilibrium. Based on the observed allele frequencies, the expected genotype counts were approximately 38.4 AA, 50.2 AG, and 16.4 GG, whereas the observed counts were fifty-nine AA, nine AG, and thirty-seven GG (Figure 3). This discrepancy was driven mainly by a pronounced deficit of heterozygous dogs.

The genotype distribution in the present cohort was highly consistent with the CKCS population reported by Meurs et al., in which forty-three of seventy-three dogs were homozygous for the ACE polymorphism, five were heterozygous, and twenty-five were the homozygous wild type [34]. In the present cohort, the corresponding distribution was fifty-nine AA, nine AG, and thirty-seven GG. A direct comparison of genotype distributions between the present cohort and the Meurs et al. CKCS cohort did not reveal a significant difference. The prevalence of variant-positive dogs was also nearly identical: 64.8% in the present cohort compared with 65.8% in the Meurs et al. cohort. This close agreement supports the reproducibility of a high prevalence of the ACE variant in CKCS populations affected by, or predisposed to, myxomatous mitral valve disease.

**Figure 4:**
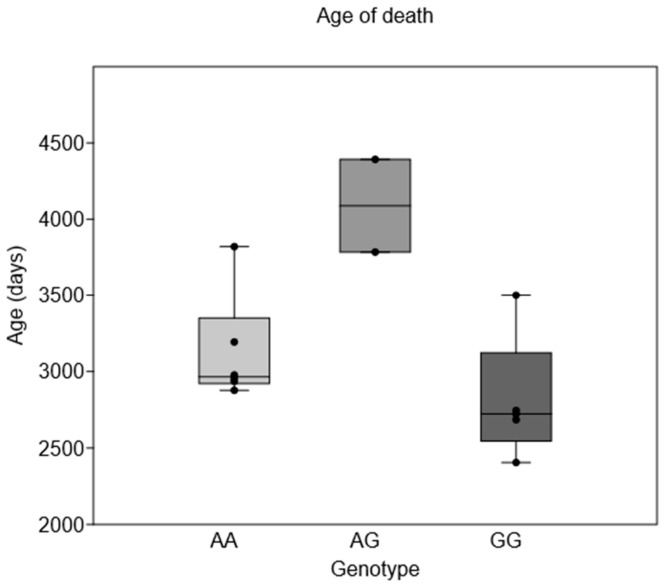
Box plot showing median age at death in days by genotype, including maximum and minimum values and 25–75 percentile. Kruskal–Wallis statistic = 6.538; *p* = 0.0380.

Kruskal–Wallis or Mann–Whitney tests, depending on the number of genotype groups available for each endpoint, did not show a statistically significant association between genotype and age at which stages B2 or C or death occurred. Analyses stratified by gender also did not show statistically significant genotype-related differences in age at disease progression endpoints. In the Mann–Whitney test (for AA and GG homozygous dogs; for no homozygous dogs was the day of entry into stage B2 recorded), no statistically significant difference was observed between genotype and age of entry into stage B2. For the entire group, *p* = 0.4725; for females, *p* = 0.8571; and for males, *p* = 0.6828. In the Kruskal–Wallis test, no statistically significant difference was observed between genotype and age of entry into stage C. For the entire group, *p* = 0.0734; for females, *p* = 0.2019; and for males, *p* = 0.0542. In the Kruskal–Wallis test, there was a statistically significant difference between genotype and age at death. For the entire group, *p* = 0.0380; for males, *p* = 0.0458; and due to the fact that only four females (two AA females, one AG female, and one GG female) reached this endpoint, statistical analysis was not performed for females (Figure 4). A Kaplan–Meier survival analysis was performed to compare time to death among genotype groups. Dogs without a recorded death event were treated as censored at the last available follow-up date. Survival curves were compared using the log-rank Mantel–Cox test. No significant difference in survival was observed between AA, AG, and GG genotype groups (Chi square = 0.1508, *p* = 0.9274).

## 4. Discussion

In the present CKCS cohort, the most plausible biological and population-level explanation of the marked deviation from the Hardy–Weinberg equilibrium is population stratification combined with breed-line structure. The Wahlund effect describes an apparent excess of homozygotes and a deficit of heterozygotes when genetically differentiated subpopulations are analyzed together as a single population. This is relevant for purebred dogs because, even within one breed, animals may originate from different breeding lines, kennels, or founder subpopulations with different allele frequencies [38,51,53].

A second likely explanation is inbreeding and the relatedness among sampled animals. Purebred dog populations are known to have restricted effective population sizes and increased homozygosity as a consequence of closed studbooks, founder effects, and intensive artificial selection. Calboli et al. showed substantial inbreeding in several purebred dog breeds and estimated low effective population sizes in most of the breeds analyzed, while Parker et al. demonstrated strong genetic structuring among purebred dogs, with approximately 30% of genetic variation explained by breed differences [38,41,52].

This issue is particularly relevant in Cavalier King Charles Spaniels because CKCS are a highly selected breed with strong predisposition to MMVD. Bionda et al. used genomic analyses, including runs of homozygosity, in CKCS with early-onset MMVD and controls, indicating that homozygosity patterns and breed-specific genomic structure are relevant in studies of MMVD genetics in this breed [54].

A technical explanation should also be considered. Genotyping error, including allele dropout, the misclassification of heterozygotes as homozygotes, poor read depth, mapping problems, or allele-balance distortion, can produce apparent deviations from HWE. This is why HWE testing is commonly used as a quality-control step in genetic studies, although deviation from HWE is not by itself proof of genotyping error. In our opinion, a technical error is the least likely factor in this case. We found no evidence that the observed deviation from expected genotype frequencies was due to methodological errors. All samples were double-checked, and genotyping was validated at the genetics laboratory level and by independent evaluation of the results by the authors. The concordance of genotyping results was 100%, indicating that technical error is an unlikely source of the observed differences [55,56].

However, the influence of selection bias cannot be completely ruled out as the study group was not a random sample of the entire CKCS population but included dogs that met specific inclusion criteria and had complete clinical records and biological material for genetic testing. Furthermore, the animals’ eligibility for the study was not related to the genotype being analyzed, as it was unknown at the recruitment stage. In our opinion, a more likely explanation for the observed deviation from the Hardy–Weinberg equilibrium is the genetic specificity of the study population. Cavalier King Charles Spaniels constitute a breed with a limited gene pool, which may be affected by founder effects and intentional or unintentional inbreeding. These factors can lead to deviations from the Hardy–Weinberg equilibrium despite properly performed genotyping. Because we did not have complete pedigree data for all animals, it was not possible to comprehensively assess the degree of relatedness between patients.

The deviation from the Hardy–Weinberg law certainly requires extending the analysis in terms of the occurrence of the variant depending on the closeness of the relationship of individuals—it is possible that such a distribution of the variant in the population results from the intentional or unintentional inbreeding of individuals of this dog breed [47,48,49].

Although previous studies have demonstrated a functional effect of the canine ACE variant on circulating ACE activity and pharmacodynamic responses related to the RAAS system, this present study did not demonstrate a strong significant association between genotype and the clinical progression of MMVD [32,33,34,35]. While a statistically significant difference was obtained between the age of death of animals with different genotypes, this significance was not reflected in the Kaplan–Meyer survival analysis. This apparent discrepancy is biologically plausible. The available evidence suggests that the ACE variant is associated with lower serum ACE activity, including in Cavalier King Charles Spaniels with MMVD, and may contribute to RAAS suppression or aldosterone elevation during ACE inhibitor treatment; however, these findings do not necessarily imply that the variant is an independent determinant of valve disease progression [32,33,34,35]. MMVD progression is a complex, multifactorial process driven primarily by age- and breed-related remodeling of the valve extracellular matrix, changes in mechanical loading, serotonergic and TGF-β-related pathways, and secondary neurohormonal activation. Therefore, genotype-related differences in ACE activity may modify neurohormonal tone or treatment response but may not be strong enough to clearly determine the timing of progression from B1 to B2, C, or death [37].

A potential factor that requires further investigation is the difference in RAAS activity in stage B2, a period in which treatment with ACE inhibitors or spironolactone is not typically implemented. Perhaps identifying higher variant-dependent RAAS activity in the preclinical stage and implementing RAAS-inhibiting therapy will improve the efficacy of MMVD treatment in dogs.

Our study has limitations. Due to the lack of access to full pedigrees, the exact relationship status of all dogs examined is not fully known, which could potentially have contributed to the reduced genetic diversity of the study group. This is, however, unlikely due to the two-center nature of the study. One of the centers is a university, a nationally recognized institution receiving dogs from across Poland. In the studied cohort, the relationship was confirmed only for four individuals—two dogs were siblings and two dogs had parent–child status, which constituted 3.8% of the studied population; for the remaining dogs, the alleged lack of relationship was based on the owners’ declaration (96.2%).

A second factor potentially limiting the statistical power of the conducted analyses might be the relatively small number of dogs that reached stage B2 of the disease and later, as well as the percentage of patients with whom contact was lost. While the number of patients reaching a given stage of the disease is purely a random factor, the percentage of patients with whom contact was lost at 21.9% is acceptable compared to other clinical studies in which higher attrition values were achieved and the research was considered valid [57,58].

An additional potential limitation of this study may be the use of two DNA sequencing techniques. However, we believe that the use of two genotyping methods did not affect the study results or introduce bias. The NGS results were verified using the Sanger method, which is considered the reference method for validating genetic variants. No discrepancies were found between the results obtained using both methods. Furthermore, all analyses were performed using the same reference sequence and the same definition of the variant being analyzed [59].

## 5. Conclusions

In summary, our study revealed a similar frequency of the ACE gene variant rs850683722 compared to the studies published by Meurs et al [34]. Carrier status of this variant in our cohort has no statistically significant effect on the course of MMVD determined by the age at onset of the disease stages. A statistically significant difference in the age of death in the absence of a statistically significant difference in the Kaplan–Meier survival curves is not sufficiently strong evidence to clearly identify this variant as significantly influencing the course of MMVD. Therefore, the assessment for variant carrier status does not seem to be a reliable genetic marker suggesting a predisposition to a more severe or less severe course of MMVD in the Polish CKCS population. The potential impact of the variant on RAAS activity in stage B2 requires evaluation in further studies.

## Figures and Tables

**Table 1:** Summary of the clinical status of dogs participating in the study as of 25 May 2026.

ACVIM Stage/Endpoint	A	B1	B2	C	Death	Lost to Follow-Up
Number of dogs	0	49	11	9	13	23

## Data Availability

The NGS sequencing data table, raw NGS and Sanger sequences, echocardiographs, electrocardiographs, blood pressure, and other clinical data on the dogs participating in the study are available from the corresponding authors upon reasonable request.

## Supplementary Materials

The following supporting information can be downloaded at: https://www.mdpi.com/article/10.3390/ani16131956/s1, Table S1: Summary table containing basic data on dogs participating in the study—gender, date of birth, genotype, and ACVIM stage on the days of recruitment to the study and as of 25 May 2026.
